# 

**Published:** 1888-11

**Authors:** 


					﻿NOVEMBER, 1888.
OLD SERIES
Vol. XXV
NEW SERIES
VoT V.—No. 9.\
<7 1855 O

HEDIGAL^wW
pJOURNAfc

-C/fcv-U^^V ">r/■ •&-

W.F.WESTMORELAND.M Dj
H.V.M.MILLER.M.D.LL.D.
©UBIJSHED BY JAMES P.HARR1SON&.CQS
!P __________ tflT LANTA,GA. A£
SUBSCRIPTION' 13.00 A YEAR.
				

